# Differential expression and prognostic value of the chemokine receptor CXCR4 in bronchopulmonary neuroendocrine neoplasms

**DOI:** 10.18632/oncotarget.3242

**Published:** 2014-12-30

**Authors:** Daniel Kaemmerer, Christiane Reimann, Elisa Specht, Ralph M. Wirtz, Manal Sayeg, Richard P. Baum, Stefan Schulz, Amelie Lupp

**Affiliations:** ^1^ Department of General and Visceral Surgery, Zentralklinik Bad Berka, Bad Berka, Germany; ^2^ Department of Pharmacology and Toxicology, Jena University Hospital, Jena, Germany; ^3^ Stratifyer Molecular Pathology GmbH, Cologne, Germany; ^4^ Department of Internal Medicine, Gastroenterology and Endocrinology, Zentralklinik Bad Berka, Bad Berka, Germany; ^5^ Department of Molecular Radiotherapy and Molecular Imaging, Center for PET/CT, Zentralklinik Bad Berka, Bad Berka, Germany

**Keywords:** lung cancer, neuroendocrine neoplasm (NEN), small cell lung cancer (SCLC), CXCR4, immunohistochemistry (IHC)

## Abstract

**Introduction:**

For many tumors, the overexpression of the chemokine receptor CXCR4 is associated with increased malignancy and poor patient outcomes. However, comprehensive data for neuroendocrine neoplasms of the lung are still lacking.

**Methods:**

CXCR4 expression was evaluated in a panel of bronchopulmonary neuroendocrine neoplasms (BP-NEN) comprising typical carcinoids (n = 26), atypical carcinoids (n = 30), and small cell lung cancers (SCLC, n = 34). Samples were analyzed by immunohistochemistry using the novel monoclonal rabbit anti-human CXCR4 antibody UMB-2 and by qRT-PCR. The expression was correlated with clinical data and overall patient survival.

**Results:**

CXCR4 was predominantly localized at the plasma membrane of the tumor cells. CXCR4 was expressed with a high intensity in almost all of the 30 SCLC samples. In contrast, it was detected infrequently and with low intensity in the typical carcinoid and atypical carcinoid samples. There was a significant correlation between the immunohistochemistry and qRT-PCR data. Additionally, there was a significant negative relationship between CXCR4 expression and overall survival.

**Conclusions:**

With increasing malignancy, BP-NEN clearly differ in the extent of CXCR4 expression. As in other tumor entities, CXCR4 overexpression significantly correlates with negative patient outcome. Due to its particular high expression rate in SCLC, CXCR4 may serve as a promising new target for diagnostic and pharmacological intervention as well as for peptide receptor-based radionuclide therapy.

## INTRODUCTION

Neuroendocrine neoplasms (NEN) can be found in many organs and represent a heterogeneous group of malignancies deriving from the neuroendocrine system. Two thirds of the cases occur in the gastroenteropancreatic tract [1]. Clinically, NEN are commonly classified into functioning and non-functioning tumors, depending on the hormone secretion [2]. One quarter of all NEN are located in the lung. The growing importance of bronchopulmonary neuroendocrine neoplasms (BP-NEN) is further underlined by a distinct increase in the incidence of BP-NEN in recent years [3].

BP-NEN can be classified into two subgroups with four different entities. Typical carcinoids (TC) and atypical carcinoids (ATC) are well-differentiated neuroendocrine tumors of the lung. Small cell lung cancers (SCLC) and large cell neuroendocrine carcinomas (LCNEC) are undifferentiated [4].

TC are characterized by a very low proliferation rate and a low rate of malignancy. ATC have an intermediate rate of malignancy: ATC are less aggressive than SCLC but are more destructive than TC. The clinical behavior of ATC depends on the height of the mitotic index and the resulting proliferation rate, which positively correlates with aggressive behavior [4]. Hence, metastases are found more frequently in ATC than in TC, and distant metastases are often already present at the time of ATC diagnosis [5, 6]. Finally, SCLC and LCNEC are very aggressive BP-NEN and are usually associated with a poor prognosis [5, 7].

For TC and ATC, the first-line therapy is curative surgery, leading in general to prolonged long-term survival [8, 9]. In contrast, surgery is recommended for few patients with SCLC and only for those with a limited disease status [7, 10]. In most cases, patients with SCLC display an advanced tumor stage, and systemic chemotherapy with initially high response rates is the standard therapy. The overall 5-year survival rate among patients with SCLC is limited to 5–10% due to early recurrence, whereas the 5-year and 10-year survival rates are 80–90% among patients with TC and 50–70% among patients with ATC [5, 7].

The chemokine receptor CXCR4 is a seven transmembrane G-protein coupled receptor [11]. Physiologically, CXCR4 is expressed by different cells of the hematopoietic system (e.g., T and B lymphocytes, monocytes, and macrophages). The activation of the receptor by its natural ligand CXCL12 (SDF-1, stromal cell derived factor 1) leads to the proliferation of cells and directed migration towards the source of the ligand [12]. Hence, the CXCR4/CXCL12 axis is important for the homing and retention of stem cells and the trafficking of lymphocytes towards the sites of tissue damage or inflammation [13]. The receptor is additionally involved in many physiological processes including embryonic development, hematopoiesis, and angiogenesis [14]. The overexpression of CXCR4 has been found in at least 23 different types of tumors [15]. Hence, CXCR4 is postulated to be involved in tumor progression, metastasis, adaptation to hypoxia, and stem cell survival. Many studies have demonstrated a correlation between CXCR4 expression and tumor aggressiveness with regard to metastatic spread and limited patient overall survival (OS) [16-18]. Additionally, the expression of CXCR4 by cancer cells seems to be associated with malignancy potential and tumor recurrence. Therefore, the CXCR4/CXCL12 axis is a promising target for cancer diagnostics and therapies, which is reflected by the development and application of a large number of antagonistic CXCR4 ligands.

Plerixafor (AMD3100) has been approved by the United States Food and Drug Administration and in the European Union by the European Medicines Agency for stem cell mobilization [19]. The use of AMD3100 labeled with Gallium-68 in breast cancer-bearing mice demonstrated the potential of CXCR4 antagonists as imaging reagents on the one hand and reflects the significance of CXCR4 as a marker structure in cancer on the other hand [20]. Previously, Gourni et al. presented the first data on the new ^68^Ga-labeled high-affinity CXCR4 ligand ^68^Ga-CPCR4-2 (cyclo(D-Tyr(1)-[NMe]-D-Orn(2)-[4-(aminomethyl) benzoic acid), which is characterized by high *in vivo* stability and distinct and specific tumor accumulation [21]. The *in vivo* anti-metastatic efficacy of CXCR4 antagonists, such as TF 14016 [22], CTCE-9908 [23], and AMD3465 [24], has been demonstrated in several animal trials. Some CXCR4 antagonists have even been tested in clinical studies with the aim to treat different kinds of cancers (e.g., hematological cancers and brain tumors [25]).

In contrast to many other tumor entities, the role of CXCR4 expression has not been evaluated in typical and atypical lung carcinoids so far, and there are only limited data available on CXCR4 expression in SCLC [26, 27]. In addition, previous studies investigating the expression of CXCR4 in different tumor entities by immunohistochemistry (IHC) often yielded only cytoplasmic or nuclear staining of this membrane-bound receptor [28, 29]. Therefore, the aim of the present study was the immunohistochemical evaluation of CXCR4 expression in BP-NEN using the monoclonal rabbit anti human CXCR4 antibody UMB-2, which, in contrast to other anti-CXCR4 antibodies, leads predominantly to membranous staining of the receptor. Hence, when evaluating the staining results, only membranous staining was taken into account. Additionally, the immunohistochemical findings were verified at the mRNA level by qRT-PCR. Finally, CXCR4 expression as determined by IHC and qRT-PCR was correlated with patient and clinical data.

## RESULTS

### Clinical data

For all three tumor entities, the mean age of the patients was similar (TC: 59.9±14.9, ATC: 58.3±15.3, SCLC: 59.9±9.4 years; Table 3). The ages of the patients with TC or ATC were more variable than those of the patients with SCLC. Accordingly, the minimum age of the patients with TC or ATC was 18 years, whereas that of the patients with SCLC was 43 years. Most of the patients with TC were female (19 females vs. 7 males), whereas most of the patients with SCLC were male (22 males vs. 12 females). Men and women were equally distributed among the patients with ATC (15 males vs. 15 females). Supplementary Table 1 presents the available TNM stages. In the TC and SCLC group only primary tumors were included. Within the ATC primary tumors and 3 metastases were enclosed.

**Table 1:** Immunoreactive score (IRS) of immunohistochemical staining

Percentage of positive cells	Intensity of staining	IRS
0 (no positive cells)	0 (no reaction)	0–2 (negative)
1 (< 10% positive cells)	1 (mild reaction)	3–4 (mild)
2 (10–50% positive cells)	2 (moderate reaction)	5–8 (moderate)
3 (50–80% positive cells)	3 (intense reaction)	9–12 (strongly positive)
4 (> 80% positive cells)		

**Table 2:** ΔCT values of CXCR4 mRNA levels and division into quartiles

ΔCT values	mRNA level quartiles
26.00–29.49	0
29.50–32.99	1
33.00–36.49	2
36.50–40.00	3

**Table 3:** Patient data

	TC	ATC	SCLC
Sex [N]			
Female	19	15	12
Male	7	15	22
Age [years]			
Median	62.8	62.1	59.6
Mean	59.9	58.3	59.9
Min	18.2	18.2	43.4
Max	81.0	76.4	79.6
SD	14.9	15.3	9.4
Survival [months]			
Median	80.7	89.7	21.5
Mean	80.2	80.8	32.9
Min	3.9	3.5	0.1
Max	129.2	137.2	119.0
SD	31.7	41.2	32.8
CXCR4 expression [IRS score]			
Median	0	2	9
Mean	0.9	2.22	7.82
Min	0	0	0
Max	5	11	12
SD	1.44	2.64	4.3
CXCR4 mRNA level [ΔCT values]			
Median	34.8	35.6	37.9
Mean	34.3	35.6	37.5
Min	26.0	33.25	35.58
Max	39.45	37.45	39.35
SD	2.96	1.5	1.0

### Survival data

Overall, CXCR4 expression in BP-NEN as detected by IHC and qRT-PCR was significantly and inversely correlated with OS (IRS CXCR4 × OS: n=70, r=-0.435, p<0.001; mRNA ΔCt-value CXCR4 × OS: n=56, r= −0.455, p<0.001; Table 4). Within the tumor entity subgroups, however, no significant association between CXCR4 expression and OS were found.

**Table 4:** Correlation of CXCR4 IRS and ΔCT with overall survival

IRS / ΔCT value	Number of patients	Correlation (r)	P-value
IRS CXCR4 × OS	N=70 (TC, ATC, SCLC)	−0.435	<0.001
ΔCT value CXCR4 × OS	N=56 (TC, ATC, SCLC)	−0.455	<0.001

IRS CXCR4 × OS	N=22 (TC)	0.179	0.427
ΔCT value CXCR4 × OS	N=19 (TC)	−0.212	0.383

IRS CXCR4 × OS	N=27 (ATC)	−0.043	0.830
ΔCT value CXCR4 × OS	N=19 (ATC)	−0.284	0.238

IRS CXCR4 × OS	N=21 (SCLC)	−0.016	0.944
ΔCT value CXCR4 × OS	N=18 (SCLC)	0.278	0.264

**Figure 1:**
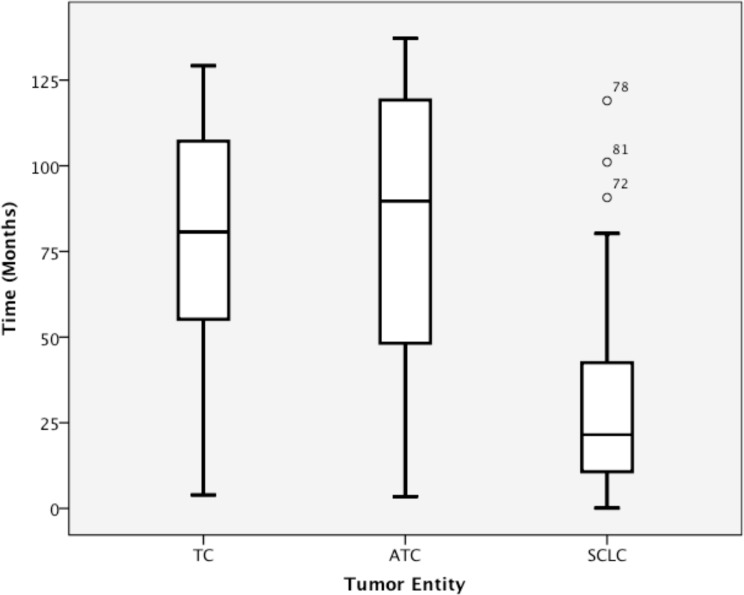
Overall survival among patients with TC, ATC, and SCLC.

The mean survival time was higher in the TC (80.2 months) and ATC (80.8 months) subgroups than in the SCLC subgroup (32.9 months; Table 3; Figure 1). The Mann-Whitney-U test revealed a significant association between tumor entity and survival, especially when the TC and ATC subgroups were combined and compared with the SCLC subgroup. There was no significant difference in OS between the TC and AC subgroups (p=0.786), whereas comparisons between the TC and SCLC subgroups and between the ATC and SCLC subgroups revealed highly significant differences (p<0.001 for each comparison).

**Figure 2:**
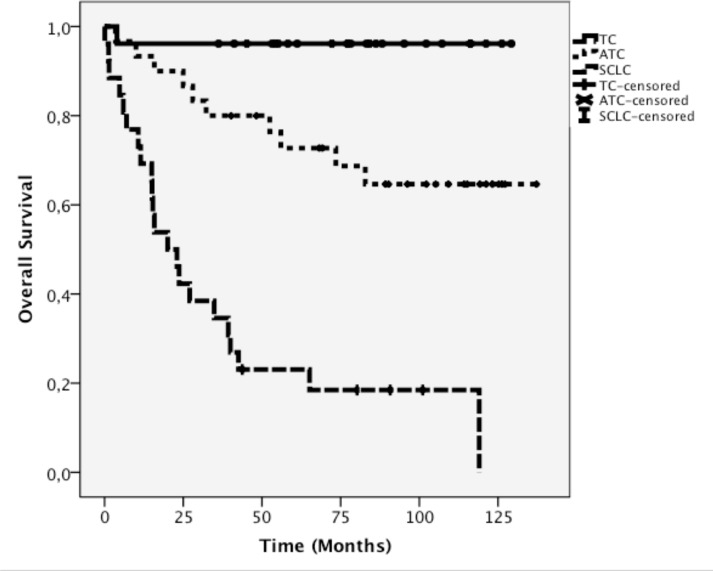
Kaplan-Meier curve of overall patient survival for TC, ATC, and SCLC.

Accordingly, the Kaplan-Meier-analysis revealed a significant difference in OS among the TC, ATC, and SCLC subgroups (log rank p<0.001; Figure 2).

### CXCR4 immunohistochemistry

**Figure 3:**
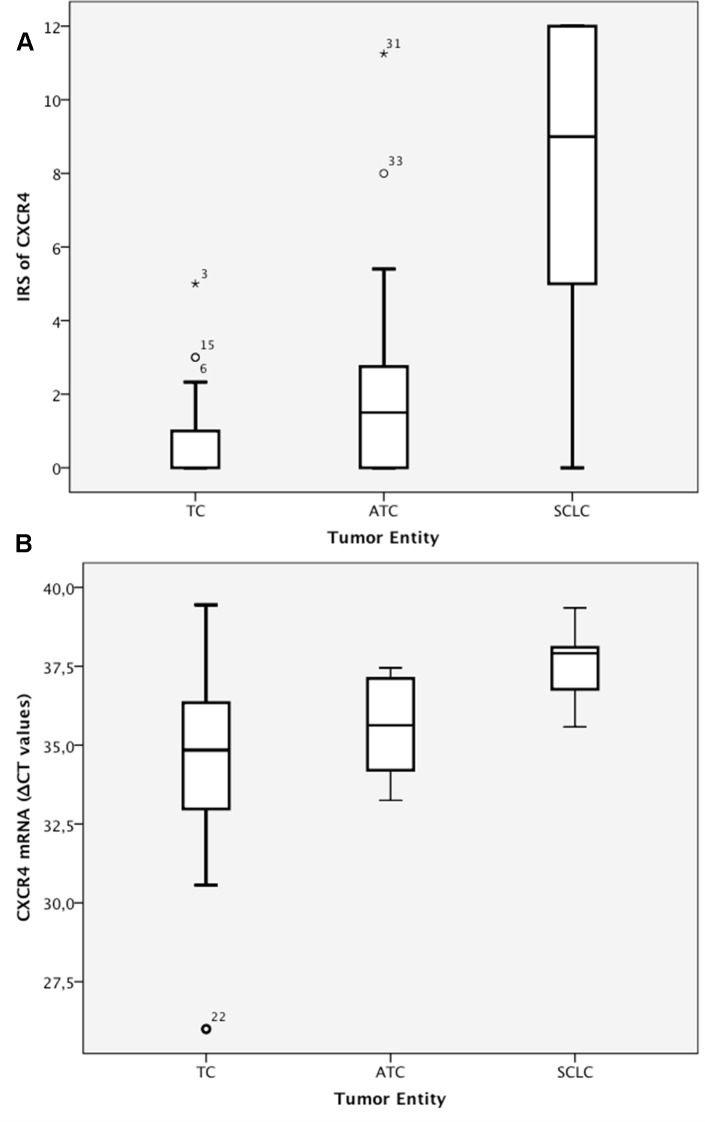
A) IRS (0–12) of CXCR4 immunohistochemistry. B) CXCR4 mRNA levels (ΔCT values).

The IRS of the CXCR4 staining differed significantly among the three tumor entities. The median IRS was 0 (range: 0–5) in the TC subgroup, 2 (range: 0–11) in the ATC subgroup, and 9 (range: 0–12) in the SCLC subgroup (Table 3; Figure 3A).

**Figure 4:**
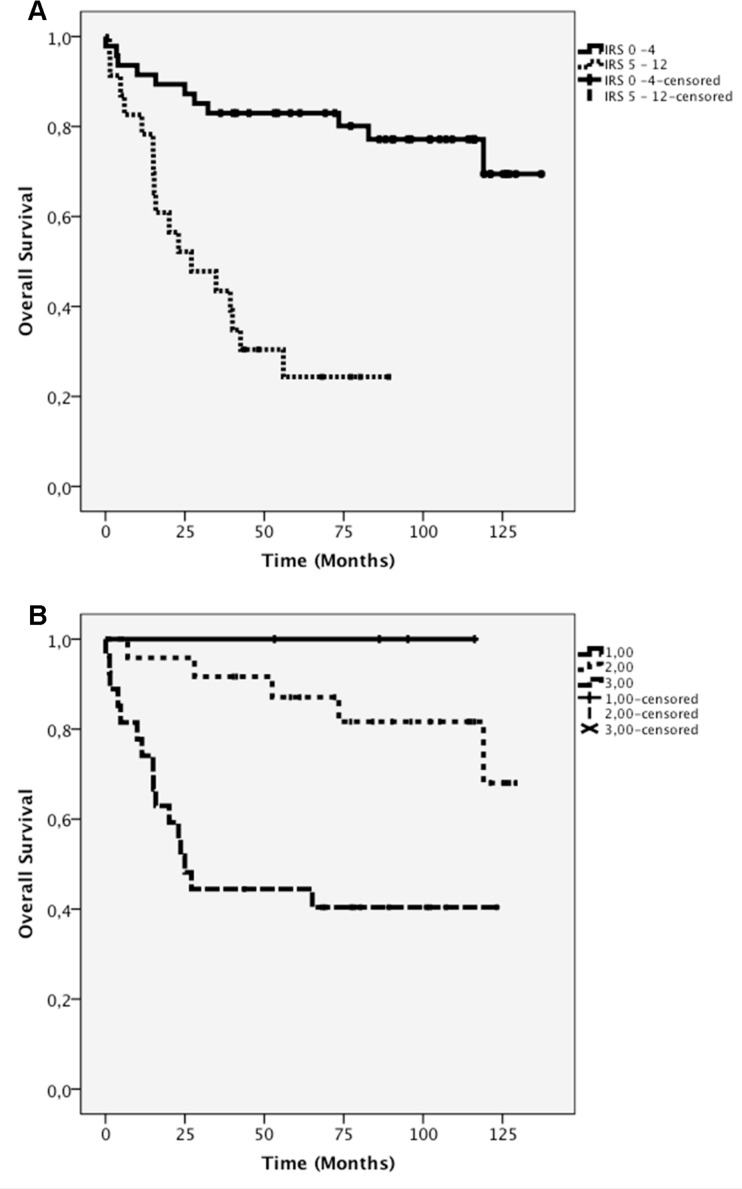
A) Kaplan-Meier survival curves for negative-tomild (IRS 0–4) and moderate-to-strong (IRS 5–12) CXCR4 expression as determined by immunohistochemistry. B) Kaplan-Meier curve of CXCR4 mRNA levels (0–3).

The receptor expression as detected by IHC was directly associated with OS. Patients with a low IRS (0–4) had significantly higher OS compared with patients with a high IRS (5–12; log-rank p<0.001). Thus, strong CXCR4 staining was directly associated with low OS (Figure 4A).

**Figure 5:**
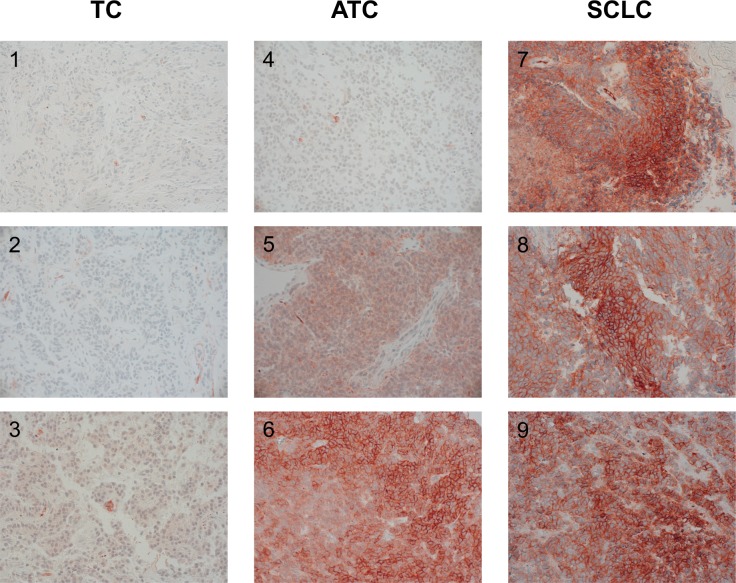
CXCR4 expression in the tumor samples of 3 patients with TC (1-3), of 3 patients with ATC (4-6), and 3 patients with SCLC (7-9), (immunohistochemistry; original magnification: 400x)

A low IRS (0–4) was observed in 21/22 patients with TC (median OS=86.2 months), whereas a high IRS (5–12) was found in only one TC patient (OS=77.2 months; Table 5). With ATC a low IRS (0–4) was noticed in 23/27 patients (median OS=96.2 months) and a high IRS (5–12) in 4/27 patients (median OS=62.1 months; Table 5). In only 3/21 of the SCLC patients a low IRS (0–4) could be detected (median OS=90.7 months), whereas a high IRS (5–12) was seen in 18/21 of the cases (median OS=17.9 months; Table 5). Figure 5 demonstrates the typical immunohistochemical images of CXCR4 expression in TC, ATC and SCLC.

**Table 5:** CXCR4 expression at the protein and mRNA levels and overall survival in TC, ATC and SCLC

Immunohistochemistry TC			
IRS 0–12	N = 22	Events	Median survival [months]
IRS 0–4	N=21	1	86.2
IRS 5–12	N=1	0	77.2
CXCR4-mRNA Quartiles TC			
PCR Quartiles 0–3 (ΔCT values)	N=19	Events	Median survival [months]
0 (26.00–29.49)	N=1	0	126.2
1 (29.50–32.99)	N=4	0	90.7
2 (33.00–36.49)	N=10	0	80.7
3 (36.50–40.00)	N=4	1	77.7
CXCR4-Immunohistochemistry ATC			
IRS 0–12	N=27	Events	Median survival [months]
IRS 0–4	N=23	7	96.2
IRS 5–12	N=4	1	62.1
CXCR4-mRNA Quartiles ATC			
PCR Quartiles 0–3 (ΔCT values)	N=19	Events	Median survival [months]
2 (33.00–36.49)	N=12	2	100.7
3 (36.50–40.00)	N=7	2	69.2
CXCR4-Immunohistochemistry SCLC			
IRS 0–12	N=21	Events	Median survival [months]
IRS 0–4	N=3	2	90.7
IRS 5–12	N=18	16	17.9
CXCR4-mRNA Quartiles SCLC			
PCR Quartiles 0–3 (ΔCT values)	N=18	Events	Median survival [months]
2 (33.00–36.49)	N=2	2	63.0
3 (36.50–40.00)	N=16	13	17.9

### Gene expression data

In the PCR analysis, the ΔCT values differed significantly between the SCLC subgroup on the one hand (median=37.9, range: 35.58–39.35) and the TC and ATC subgroups on the other hand (p<0.001). In contrast, the median mRNA level was not significantly different between the TC subgroup (median=34.8, range: 26.0–39.45) and the ATC subgroup (median=35.6, range: 33.25–37.45; p=0.102; Table 3; Figure 3B).

In the TC subgroup, the two lower quartiles (0 and 1) of the CXCR4 mRNA level comprised only a few patients (quartile 0: 1 of 19 ≙ 5.3%; quartile 1: 4 of 19 ≙ 21%). The majority of the patients in the TC subgroup had moderate mRNA levels (quartile 2: 10 of 19 ≙ 52.6%), and only a few displayed high mRNA levels (quartile 3: 4 of 19 ≙ 21%). Only one patient in the TC group had died, and that patient's mRNA level was located within quartile 3 (≙ 25%; Table 5).

In the ATC subgroup, the majority of the patients had moderate mRNA levels (quartile 2: 12 of 19 ≙63%), but some patients showed also high mRNA levels (quartile 3: 7 of 19 ≙ 37%; Table 5). In quartiles 2 and 3, respectively, two patients had died (quartile 2: ≙ 16.7 %, quartile 3: ≙ 28.6%). The median OS time was 101 months in quartile 2 and 69 months in quartile 3 (Table 5).

In contrast, nearly all of the patients in the SCLC subgroup exhibited high mRNA levels (quartile 3: 16 of 18 ≙ 89%), while some had moderate mRNA levels (quartile 2: 2 of 18 ≙ 11%; Table 5). The majority of the patients with SCLC in quartile 3 had died (n=13 ≙ 81%), and the median OS time in that quartile was only 17.9 months. Both of the patients in quartile 2 had died (≙ 100%), but the median OS time in that quartile amounted to 63 months (Table 5).

The data of quartile 0 were excluded from the comparative survival analysis due to the presence of one patient only. The survival curves corresponding to the low and moderate CXCR4 mRNA expression levels (quartiles 1 and 2) were not significantly different from each other (log-rank p=0.457). There was also no significant difference between the survival curves of quartiles 1 and 3 (log-rank p=0.064), whereas a significant difference was found between the survival curves of quartiles 2 and 3 (log-rank p=0.001; Figure 4B).

### Correlation between the immunohistochemistry and qRT-PCR data

Among all the patients, the CXCR4 expression as detected by IHC displayed a significant correlation with the CXCR4 mRNA level (n=51; r=0.691; p<0.001). Within the three tumor subgroups, however, the results were different. In the TC subgroup, there was no correlation between the CXCR4 protein level and the mRNA level (n=17, r=0.109, p=0.676). In the ATC subgroup, a strong trend for an association between the protein and mRNA levels was noticed (n=17, r=0.472, p=0.056) and, finally, in the SCLC subgroup a significant correlation between the CXCR4 protein and mRNA levels could be detected (n=17, r=0.510, p=0.037).

## DISCUSSION

### Clinical data

The mean age of the patients in each of the three bronchopulmonary tumor entities was within the sixth decade of life, which is in line with that in other studies [9, 30]. TC and ATC also occurred in younger patients, whereas SCLC only affected patients with advanced age. Additionally, the development of SCLC seems to be associated with the male gender. Because the occurrence of SCLC is correlated with cigarette smoking, one reason for the gender bias may be that cigarette smoking is still more common among males than among females. In contrast to that seen in SCLC, a correlation between smoking and the development of pulmonary carcinoids has not been proven so far [8, 31]. Madrid-Carbajal et al., for example, could not find any relationship between tobacco consumption and TC incidence [32]. In our investigation, and in contrast to the other tumor entities, the TC subgroup contained more females than males. From the literature, however, no data are available that support a higher TC incidence in women than in men [30, 33].

### CXCR4 expression and survival data

CXCR4 is widely expressed in different malignant tumor entities. Many studies have found an association between the CXCR4 expression level and fast tumor recurrence as well as poor patient outcomes [34-36].

Among the pulmonary cancer entities, CXCR4 has been shown to be highly expressed in primary and secondary lung cancers [17]. Reports on CXCR4 expression in SCLC and CXCR4 expression levels in pulmonary carcinoids are very limited [26, 27, 37].

In the present study, the CXCR4 expression in BP-NEN was analyzed at the protein level as well as the mRNA level and correlated with survival data for the first time. In TC and ATC, patient survival is not as poor as that in SCLC. CXCR4 expression as detected by IHC (IRS values) differed significantly between patients with TC and those with ATC, whereas the mRNA levels did not. One reason for that discrepancy may be that not necessarily all mRNA is translated into protein. The higher CXCR4 protein levels in ATC compared to those in TC could be responsible for the higher nodal metastasis rate, earlier recurrence, and enhanced aggressiveness of the ATC tumor entity [18, 38]. As shown in previous studies, nodal involvement seems to be a prognostic criterion for tumor recurrence [39]. Metastases and recurrence were described previously, however, for both TC and ATC [6, 39]. Our clinical survival data are congruent with those investigations. To our knowledge, there are no data sets other than our own that show the CXCR4 expression levels in TC and ATC at the protein level as well as the mRNA level. Hence, our results cannot currently be compared to those of other studies.

Our data show a significant inverse correlation in SCLC between CXCR4 expression as determined by IHC (IRS values) and mRNA levels, respectively, and OS (IRS: r=-0.435, p<0.001; mRNA: r=-0.455, p<0.001). Therefore, especially in SCLC, (besides IHC) mRNA analysis could be a useful diagnostic tool.

Hartmann et al. reported high levels of functional CXCR4 in SCLC [27]. Our data completely support those findings. Previous studies have also shown that the CXCR4 ligand CXCL12 is able to induce integrin activation, resulting in increased tumor cell adhesion and metastasis in SCLC. Additionally, CXCR4 seems to be involved in tumor-stroma interactions, thus leading to protection of the SCLC tumor cells from chemotherapy-induced apoptosis. Accordingly, co-treatment with cytotoxic drugs and CXCR4 inhibitors has been recommended [26, 27].

As shown in Figure 4, survival was directly associated with the IHC data and the CXCR4 mRNA level. In our study, increased levels of both CXCR4 protein and CXCR4 mRNA led to decreased patient survival. These findings correspond to the results of many other studies, which demonstrated a relation between CXCR4 expression and poor patient survival [38, 40, 41].

SCLC and ATC with mild or strong CXCR4 expression differed mainly in OS, although not significantly. With a higher number of cases, that result would likely be statistically significant. Overall, our findings suggest a strong correlation between the CXCR4 expression level (as measured by IHC and mRNA level) and OS.

### CXCR4 immunohistochemistry and PCR data

With respect to the detection of CXCR4 expression by means of IHC, the specificity of the rabbit monoclonal antibody UMB-2 has been shown in different tumor entities [42]. Also, IHC data based on highly sensitive monoclonal antibodies and PCR data have previously been shown to be comparable [43].

Generally, our IHC and PCR data were significantly correlated with each other (r=0.691, p<0.001), demonstrating a strong interrelationship between the results of the two methods. With regard to the tumor subgroups, the correlation between the IHC data and the PCR data was only significant in the SCLC subgroup; so the correlation may only exist if the values are high enough.

### CXCR4-based imaging and treatment

**Figure 6:**
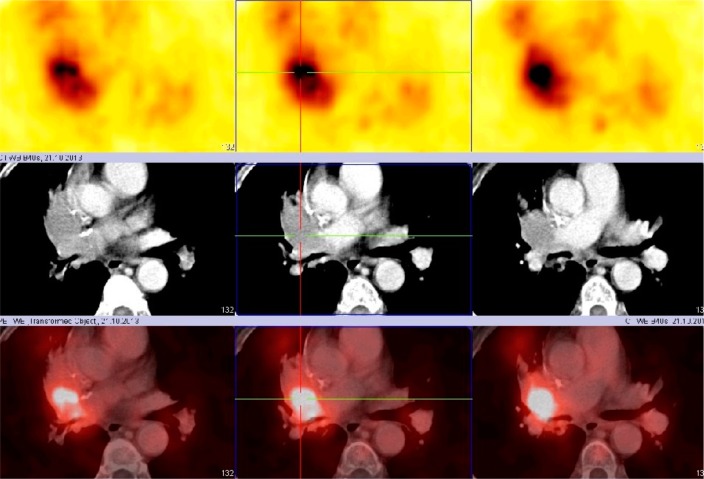
^68^Ga-CPCR4-2 PET/CT (transversal images): local recurrence of an centrally localized SCLC (upper panel: PET scan, middle panel: CT scan, lower panel: PET + CT fusion image).

Receptor-targeted imaging is performed by means of single photon emission computed tomography (SPECT) as well as positron emission tomography (PET) with many different tracers (e.g., Indium-111, Iodine-125, Copper-64, or Gallium-68). A general problem concerning imaging techniques is the accumulation of the used reagent in the liver and kidney. The long synthesis times and the low radiochemical yield when using In-111 or I-125 may cause problems [44]. The usefulness of the CPCR4-2 peptide labeled with Gallium-68 was initially reported by Gourni et al. [21]. The first clinical experience with ^68^Ga-CPCR4-2 (prepared in the radiopharmacy of our hospital) PET/CT imaging was very encouraging and demonstrated the clinical applicability and feasibility of using that molecular imaging technique for confirming CXCR4 expression in patients with SCLC; this information can then be used to select patients with high CXCR4 expression for radiopeptide therapy according to the theranostics principle (Figure 6).

The efficacy of AMD3100 as a CXCR4 antagonist has been proven in many studies for many different tumor types [45, 46]. Other antagonistic CXCR4 ligands (e.g., TF14016) have also demonstrated suppression of metastases and anti-angiogenic potential in SCLC [22]. Our IHC and PCR data verified the high CXCR4 expression levels in human SCLC reported previously. In accordance with previous studies, our data provide strong evidence for antagonistic CXCR4 ligands as a promising therapy option in SCLC and highly proliferative ATC [22, 47]. Additional experimental studies are necessary, however, to further substantiate that point of view. In a next step an own retrospective study with a larger number of SCLC cases is planned to further investigate the CXCR4 expression with regard to OS. More data on CXCR4 expression in SCLC are necessary to underline the prognostic relevance in SCLC treatment.

## CONCLUSIONS

Bronchopulmonary neuroendocrine tumors (TC, ATC, and SCLC) clearly differed in their CXCR4 expression levels. SCLC displayed very high CXCR4 protein and mRNA expression intensities. TC and ATC, in contrast, exhibited lower or nonexistent CXCR4 expression levels. We showed that increasing CXCR4 protein levels as well as mRNA levels were directly associated with limited OS. Our CXCR4 IHC and PCR data correlated significantly with each other. Highly proliferative ATC and SCLC seem to be suitable and very promising targets for (additional) CXCR4-based imaging as well as for peptide receptor based radionuclide therapy or pharmacotherapy.

## MATERIALS and METHODS

### Patients and clinical data

Between 2002 and 2011, 1385 patients with lung cancer were operated on in the Zentralklinik Bad Berka and evaluated for bronchopulmonary tumors. Fifty-eight patients with TC and ATC were evaluated. Additionally, 35 patients with histologically proven SCLC who had undergone surgery were chosen randomly from the years 1998–2002. All tumor samples were obtained as paraffin-embedded tissue specimens from the archive of the Laboratory for Pathology and Cytology, Zentralklinik Bad Berka, Germany. Three specimens (2 ATC and 1 SCLC) were excluded from the analysis because of staining artifacts.

All clinical data (age, sex, first diagnosis, follow-up, date of death, and OS) were selected from the patients records and supplemented by the local tumor registry from the Tumorcenter, Erfurt, Germany. Additionally, all TC and ATC samples were histopathologically re-evaluated by a lung-experienced pathologist. The current classification system of BP-NEN is based on two grading systems according the WHO / IASLC (International Association for the Study of Lung Cancer) guidelines [48]. Finally, 26 TC, 30 ATC, and 34 SCLC samples were included in our study. The study was approved by a local ethical committee.

### CXCR4 antibody

The rabbit monoclonal anti-CXCR4 antibody UMB-2 was obtained from Epitomics (Burlingame, CA). The antibody was generated against the amino acid sequence KGKRGGHSSVSTESESSSFHSS, which corresponds to residues 331–352 of the C-terminal tail of the human CXCR4 receptor. UMB-2 was characterized extensively in previous studies [42].

### Immunohistochemistry

Four-micrometer sections were prepared from the paraffin blocks and floated onto positively charged slides. Immunostaining was performed by an indirect peroxidase labeling method as described previously [42]. Briefly, the sections were dewaxed and microwaved in 10 mM citric acid (pH 6.0) for 16 min at 600 W for antigen retrieval. Thereafter, the samples were incubated with UMB-2 at a volumetric ratio of 1:2 overnight at 4°C. Binding of the primary antibody, followed by an incubation in a biotinylated avidin-peroxidase solution (Vector ABC “Elite” kit, Vector, Burlingame, CA) and visualized using 3-amino-9-ethylcarbazole in acetate buffer (BioGenex, San Ramon, CA). The sections were then rinsed, counterstained with Mayer's hematoxylin, and mounted in Vectamount mounting medium (Vector Laboratories, Burlingame, CA).

### Evaluation of the staining

To evaluate the intensity of the staining, the immunoreactivity score (IRS) according to Remmele and Stegner was used [49]. This score enables the determination of both the intensity of the immunosignal and the percentage of cells showing positive staining. The IRS, which has values between 0 and 12, was calculated as follows: [percentage of positive cells] × [intensity of staining] = IRS. The percentage of positive cells was scored as: no positive cells (0); <10% (1); 10–50% (2); 51–80% (3); >80% (4). The intensity of the staining was scored as: no staining (0); mild (1); moderate (2); strong (3). Only cells showing membranous staining of CXCR4 were scored as positive. For the statistical analysis, the IRS data were further classified into four groups describing negative (IRS 0–2), weak-positive (IRS 3–4), moderate-positive (IRS 5–8), or strong-positive (IRS 9–12) staining (Table 1).

### Gene expression analysis by RNA isolation and quantitative reverse-transcription polymerase chain reaction (qRT-PCR)

Nineteen TC and ATC samples, respectively, as well as 22 SCLC samples were randomly picked for PCR analysis. From those samples, additional sections were stained with hematoxylin and eosin and evaluated by a certified pathologist who recorded the percentage tumor cell content in each of the samples. Prior to RNA isolation, macrodissection of the tumor areas was performed in most of the formalin-fixed paraffin-embedded (FFPE) sections designated for the qRT-PCR with <50% tumor cell content. In all cases, sufficient RNA was isolated from the FFPE specimens for qRT-PCR, as previously described [50]. From each FFPE section (4 μm thick), RNA was isolated using a standardized, fully automated method for isolating total RNA from FFPE tissue based on germanium-coated magnetic beads (XTRAKT RNA kits, STRATIFYER Molecular Pathology GmbH, Cologne, Germany). A liquid-handling robot (XTRAKT XL, STRATIFYER Molecular Pathology GmbH, Cologne, Germany) performed both the nucleic acid extraction including DNAse digestion (as described previously in detail) [51] and the subsequent aliquoting for biobanking and pipetting for the molecular assays. The method involves extraction-integrated deparaffinization and DNase I digestion steps. DNA-free total RNA was eluted with 100 μL elution buffer and stored at −80°C. The quality and quantity of RNA were checked by measuring CALM2 expression as a surrogate for amplifiable mRNA by qRT-PCR [52, 53]. One-step qRT-PCR was applied to assess CXCR4 and CALM2 mRNA expression using gene-specific TaqMan®-based assays. Forty cycles of nucleic acid amplification were applied, and the cycle threshold (CT) 200 of the target gene was identified. Normalized CT values (ΔCT) were obtained by subtracting the CT value of the housekeeping gene CALM2 from the CT value of the target gene. The RNA results were then reported as 40-ΔCT values, which correlate proportionally to the mRNA expression level of the target gene. Samples with average CALM2 CT values < 32 were considered eligible for analysis.

The expression of the target gene, as well as that of the reference gene CALM2, was assessed in triplicate by qRT-PCR using the SuperScript III PLATINUM One-Step Quantitative RT-PCR System (Invitrogen, Karlsruhe, Germany) in a Stratagene Mx3005p (Agilent Technologies, Böblingen, Germany) [52, 53]. A commercially available human reference RNA (Stratagene qPCR Human Reference Total RNA, Agilent Technologies, Waldbronn, Germany) was used as a positive control. No-template controls were assessed in parallel to exclude contamination.

In parallel to the IHC data, the ΔCT values were evenly divided into a four-level classification system: negative (0), low (1), moderate (2), and high (3) (Table 2).

### Statistics

The data were analyzed using SPSS for Windows 19.0 (IBM®, USA). After the assessment of a normal distribution, the following tests were used: Spearman's rank correlation, Kruskal-Wallis test, Mann-Whitney-U test, and Kaplan-Meier analysis of OS. Differences among survival curves were analyzed by a log-rank test. A p-value ≤0.05 was considered significant.

## SUPPLEMENTARY MATERIAL TABLE


