# Supplementary material for: Differential expression and prognostic value of the chemokine receptor CXCR4 in bronchopulmonary neuroendocrine neoplasms
Source: Oncotarget. 2014 Dec 30;6(5):3346–58. doi: 10.18632/oncotarget.3242 (PMC4413658; doi:10.18632/oncotarget.3242)
Supplement: Supplementary file 1 [file oncotarget-06-3346-s001.pdf]

Differential expression and prognostic value of the chemokine receptor CXCR4 in bronchopulmonary neuroendocrine neoplasms

Supplementary Material

Table 1 Supplement TNM classification

Specimens	Entity	T	N	M
NEN_L_1	TC	nd	nd	nd
NEN_L_2	TC	T1	N0	M0
NEN_L_3	TC	T2	N0	M0
NEN_L_4	TC	T2	N0	M0
NEN_L_5	TC	T2	N0	M0
NEN_L_6	TC	T1	N0	M0
NEN_L_7	TC	nd	N0	nd
NEN_L_8	TC	T1	N0	M0
NEN_L_9	TC	nd	N0	nd
NEN_L_10	TC	T1	N0	M0
NEN_L_11	TC	T1a	N0	M0
NEN_L_12	TC	T1a	N1	M0
NEN_L_13	TC	T1	N0	M0
NEN_L_14	TC	T1	N0	M0
NEN_L_15	TC	T1a	N0	M0
NEN_L_16	TC	nd	N0	nd
NEN_L_17	TC	nd	N0	nd
NEN_L_18	TC	T1	N0	M0
NEN_L_19	TC	T1	N0	M0
NEN_L_20	TC	T1	N0	M0
NEN_L_21	TC	T2	N0	M0
NEN_L_22	TC	nd	N0	nd
NEN_L_23	TC	T1	N0	M0
NEN_L_24	TC	nd	nd	nd
NEN_L_25	TC	nd	N0	M1
NEN_L_26	TC	T1	N0	M0
NEN_L_27	ATC	T1	N0	M0
NEN_L_28	ATC	T4	N1	M1
NEN_L_29	ATC	T4	N1	M1
NEN_L_30	ATC	T1	N0	M0
NEN_L_31	ATC	nd	N0	nd
NEN_L_32	ATC	nd	nd	nd
NEN_L_33	ATC	nd	N0	M0
NEN_L_34	ATC	T2b	N0	M1
NEN_L_35	ATC	T1	N0	M1
NEN_L_36	ATC	nd	N0	M1
NEN_L_37	ATC	T1b	N0	M0
NEN_L_38	ATC	T1	N0	M0

NEN_L_39	ATC	T2	N0	M0
NEN_L_40	ATC	T2	N0	M0
NEN_L_41	ATC	T2	N0	M0
NEN_L_42	ATC	nd	nd	nd
NEN_L_43	ATC	nd	nd	nd
NEN_L_44	ATC	T1	N0	M0
NEN_L_45	ATC	nd	nd	nd
NEN_L_46	ATC	T2	N1	M1
NEN_L_47	ATC	nd	nd	nd
NEN_L_48	ATC	T2	N1	M0
NEN_L_49	ATC	T1	N0	M0
NEN_L_50	ATC	T1	N1	M1
NEN_L_51	ATC	nd	nd	nd
NEN_L_52	ATC	nd	N0	nd
NEN_L_53	ATC	T2	N1	M0
NEN_L_54	ATC	nd	nd	nd
NEN_L_55	ATC	nd	N0	nd
NEN_L_56	ATC	T1	N0	M0
NEN_L_57	SCLC	nd	nd	nd
NEN_L_58	SCLC	nd	nd	nd
NEN_L_59	SCLC	nd	nd	nd
NEN_L_60	SCLC	nd	nd	nd
NEN_L_61	SCLC	nd	nd	nd
NEN_L_62	SCLC	nd	nd	nd
NEN_L_63	SCLC	nd	nd	nd
NEN_L_64	SCLC	nd	nd	nd
NEN_L_65	SCLC	nd	nd	nd
NEN_L_66	SCLC	nd	nd	nd
NEN_L_67	SCLC	nd	nd	nd
NEN_L_68	SCLC	nd	nd	nd
NEN_L_69	SCLC	nd	nd	nd
NEN_L_70	SCLC	nd	nd	nd
NEN_L_71	SCLC	nd	nd	nd
NEN_L_72	SCLC	nd	nd	nd
NEN_L_73	SCLC	T1b	N2	M1
NEN_L_74	SCLC	nd	nd	nd
NEN_L_75	SCLC	nd	N1	nd
NEN_L_76	SCLC	nd	nd	nd
NEN_L_77	SCLC	nd	nd	nd
NEN_L_78	SCLC	nd	nd	nd
NEN_L_79	SCLC	T2	N1	M0
NEN_L_80	SCLC	nd	nd	nd
NEN_L_81	SCLC	T2	N0	M0
NEN_L_82	SCLC	nd	N0	nd
NEN_L_83	SCLC	nd	nd	nd
NEN_L_84	SCLC	nd	nd	nd
NEN_L_85	SCLC	nd	nd	nd

NEN_L_86	SCLC	nd	N0	nd
NEN_L_87	SCLC	T2b	N1	M0
NEN_L_88	SCLC	T2	N2	M0
NEN_L_89	SCLC	nd	nd	nd
NEN_L_90	SCLC	T1	N0	M0

nd – no data
